# Association of Lycopene and Male Reproductive Health: Systematic Review and Meta-Analysis

**DOI:** 10.3390/ijms26157224

**Published:** 2025-07-25

**Authors:** Isabel Viña, Juan R. Viña

**Affiliations:** 1 IVB Wellness Lab, C/Colón 12, 46004 Valencia, Spain; isabelvinabas@gmail.com; 2 Department of Biochemistry and Molecular Biology, Faculty of Medicine, INCLIVA Institute, University of Valencia, Av. de Blasco Ibáñez, 15, 46010 Valencia, Spain

**Keywords:** lycopene, antioxidant, male, reproductive health, semen analysis, infertility

## Abstract

Lycopene, a carotenoid found in tomatoes and watermelon, has been investigated for its potential to improve male fertility through its antioxidant and anti-inflammatory mechanisms. However, evidence of its effectiveness remains inconsistent. We conducted a systematic review and meta-analysis of studies published until February 2025 in the PubMed, Scopus, Web of Science, and Medline databases. Clinical studies evaluating lycopene supplementation in relation to male fertility outcomes were included in this review. Standardized mean differences (SMDs) were calculated for the key outcomes. Four clinical studies involving 151 participants were included. Lycopene supplementation significantly improved sperm concentration (SMD 0.33, 95% CI [0.02–0.65], *p* = 0.037) and nonprogressive motility (SMD 0.45, 95% CI [0.04–0.87], *p* = 0.032). No statistically significant effects were observed on total motility, progressive motility, normal or abnormal morphology, semen volume, or DNA damage. Sensitivity analyses showed that the findings were generally robust, although publication bias and methodological heterogeneity were noted. Lycopene supplementation may offer modest benefits in improving sperm concentration and nonprogressive motility in men. However, evidence for other fertility-related outcomes is inconclusive. Larger, high-quality randomized trials are needed to confirm these findings and clarify the role of lycopene in male reproductive health.

## 1. Introduction

Infertility is clinically defined as the failure to achieve pregnancy after 12 months of regular, unprotected sexual intercourse. Up to 90% of cases of male infertility are directly or indirectly associated with a low sperm count and/or decreased sperm quality [1].

One of the two mechanisms that explain this deterioration in sperm health is oxidative stress, which damages sperm DNA, reduces motility, and alters morphology. This stress can originate both endogenously and from external factors and is especially relevant given that sperm cells are highly vulnerable to oxidative damage because of their limited antioxidant capacity. Another, less common mechanism of sperm DNA damage is nuclease-mediated cleavage, generally associated with testicular infections or torsions [2].

Environmental pollutants harm male reproductive health via oxidative stress. Lipophilic toxins, such as heavy metals and organic pollutants including pesticides, phthalates, and polycyclic aromatic hydrocarbons, accumulate in reproductive tissues, inducing reactive oxygen species (ROS) and depleting antioxidants [3]. Spermatozoa are especially vulnerable because of their high content of polyunsaturated fats, weak antioxidant systems, and limited DNA repair [4]. The resulting damage includes lipid peroxidation, protein carbonylation, and oxidative DNA lesions such as 8-OHdG, even in normozoospermic men [5]. Endocrine disruption further impairs testosterone production and spermatogenesis [6]. Men in polluted regions show higher sperm DNA fragmentation and reduced fertility [7]. This emerging understanding highlights the critical need for antioxidant interventions that can specifically protect sperm DNA integrity from environmental oxidative assault.

Lycopene, a carotenoid responsible for the red pigmentation of tomato and watermelon, has aroused a growing interest as a natural antioxidant agent in the context of male reproductive health. Its chemical structure, with 11 double conjugated bonds (compared with 10 in lutein or 9 in beta-carotene), gives it a higher capacity to neutralize reactive oxygen species such as singlet oxygen [8]. Unlike other antioxidants such as vitamin E, lycopene does not need to be recycled to exert its action, which further enhances its effectiveness in tissues sensitive to oxidative damage, such as the testicles. Lycopene has proven to be able to specifically reduce oxidative damage in sperm, which, as we have previously mentioned, is associated with infertility in men. Thus, Moslemi et al. found that daily lycopene supplementation for a period of time up to 12 months improves parameters such as sperm concentration and even outcomes such as pregnancy [9]. Lycopene benefits male reproductive health through antioxidant and nonoxidative mechanisms. It activates Nrf2, upregulating antioxidant enzymes in testicular tissue, and modulates pathways vital to spermatogenesis, including cyclic adenosine monophosphate (cAMP) and protein kinase A activity [10]. Lycopene also reduces inflammation by lowering TNF-α and IL-6 levels [11] and supports mitochondrial function through peroxisome proliferator-activated receptor-γ coactivator-1α (PGC-1α) activation [12]. The bioavailability of lycopene is significantly influenced by several factors that must be considered to achieve optimal therapeutic efficacy. Lycopene absorption is enhanced by the simultaneous consumption of dietary fats, as this lipophilic carotenoid requires micelle formation for intestinal uptake, but is inhibited by the presence of dietary fiber and competing carotenoids, such as β-carotene [13]. Individual factors affecting absorption include genetic polymorphisms in beta-carotene 15,15′-oxygenase (BCO1) and scavenger receptor class B type I (SR-BI), which can result in up to 10-fold differences in plasma lycopene concentrations following identical supplementation [14]. Age, smoking status, and concurrent medications, particularly cholesterol-lowering drugs, also significantly affect lycopene bioavailability and tissue accumulation [15].

However, existing studies are limited in number, heterogeneous in design, and vary in the lycopene dose and duration of supplementation. A comprehensive synthesis of the current evidence is lacking, particularly regarding the effects of lycopene on sperm concentration, motility, morphology, and DNA integrity in men with or without infertility.

Therefore, this systematic review and meta-analysis aimed to evaluate the effects of lycopene supplementation on semen parameters and markers of male fertility, including sperm concentration, motility (total, progressive, and nonprogressive), morphology, semen volume, and DNA damage, by synthesizing evidence from clinical trials to customize the recommendation that are made in the therapeutic field of fertility.

## 2. Results

### 2.1. Study Selection

**Figure 1:**
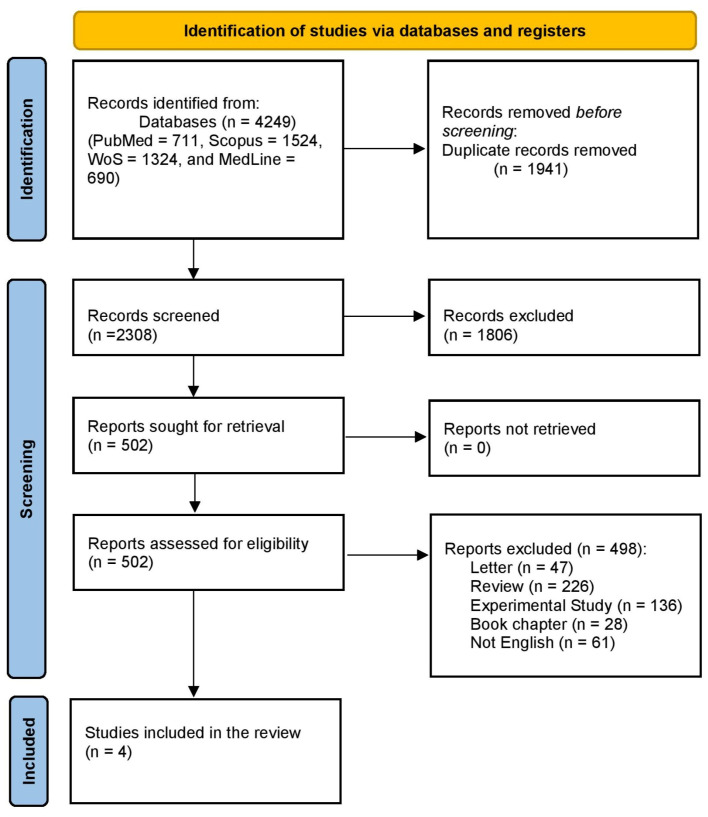
PRISMA flow chart.

A literature search through PubMed, Scopus, WOS, and Medline revealed 4249 articles, 1941 of which were duplicates. Title and abstract screening were performed on 2308 articles, of which 1806 were excluded. Then, we checked the full text of 502 studies in the full-text screening stage. Finally, four studies were included in this systematic review and meta-analysis. The study flow diagram is shown in Figure 1.

### 2.2. Summary of the Characteristics of the Included Studies and Patients

A total of four clinical studies were included in this review [16,17,18,19], comprising 151 participants across various geographic settings, namely the United Kingdom, Iran, Japan, and India. Study designs varied, with three randomized clinical trials (two of which were double-blind and placebo-controlled) and one single-arm trial. The patient populations included healthy individuals and those with primary or secondary infertility or idiopathic infertility.

Lycopene supplementation ranged in dose from 2 mg/day to 30 mg/day, administered over a duration of 3 months in all studies. The form of administration included daily or twice-daily oral doses. Williams et al. (2020) administered lycopene at a dose of 14 mg/day (7 mg twice daily) to healthy participants over three months [16]. Nouri et al. (2019) provided 25 mg/day, along with a second unspecified dose, to men with primary or secondary infertility [17]. Yamamoto et al. (2017) used a daily dose of 30 mg in infertile men [18], whereas Gupta et al. (2002) administered 2 mg twice daily (totaling 4 mg/day) to individuals with idiopathic infertility [19].

The mean age of the included patients ranged from 23.3 ± 2.58 years [16] to 38.1 ± 1.76 years [18]. Where reported, body mass index (BMI) values ranged between 23.5 ± 3.11 and 26.94 ± 1.57, indicating participants were generally in the normal to slightly overweight range. Smoking status was inconsistently reported, with the highest prevalence at 42.1% in the control group [17].

All studies reported a 3-month follow-up period. Total lycopene exposure was generally consistent across studies with daily intake ranging from 14 mg/day to 30 mg/day, except for Gupta et al. (2002) [19], which administered a lower total dose of 4 mg/day. All studies were consistent in their intervention duration and focus on lycopene’s effect in either healthy or infertile male populations. More details were presented in Table 1.

### 2.3. Primary and Secondary Outcomes

#### 2.3.1. Association Between Serum Lycopene and Sperm Concentration

**Figure 2:**
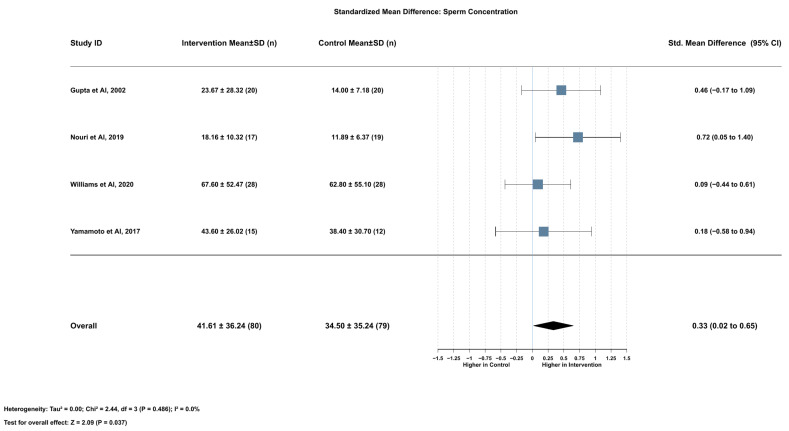
Lycopene supplementation’s effect on sperm concentration compared with control interventions [16,17,18,19].

Lycopene supplementation significantly increased sperm concentration compared with control interventions (SMD = 0.33, 95% CI [0.02 to 0.65], *p* = 0.037). This analysis encompassed four studies with a total of 159 participants (80 in the intervention group and 79 in the control group). The heterogeneity assessment yielded low statistical variation between studies (I^2^ = 0.0%), supporting the use of a fixed effects model for this outcome (Figure 2).

Leave-one-out sensitivity analyses demonstrated the overall robustness of this finding, with the effect size remaining relatively stable across different permutations. When removing Nouri et al. (2019) [17], the effect size decreased slightly and lost significance (SMD = 0.23, 95% CI [−0.13 to 0.58], *p* = 0.211), while the strongest effect emerged when removing Williams et al. (2020) [16] (SMD = 0.47, 95% CI [0.08 to 0.87], *p* = 0.018). Notably, all sensitivity analyses maintained minimal heterogeneity (I^2^ ranging from 0.0% to 11.0%), further supporting the consistency of the finding (Supplementary Figure S1). The funnel plot for sperm concentration SMD exhibited reasonable symmetry, and Egger’s test confirmed the absence of significant publication bias (z = 0.71, *p* = 0.481) (Supplementary Figure S2).

#### 2.3.2. Association Between Serum Lycopene and Total Sperm Motility

**Figure 3:**
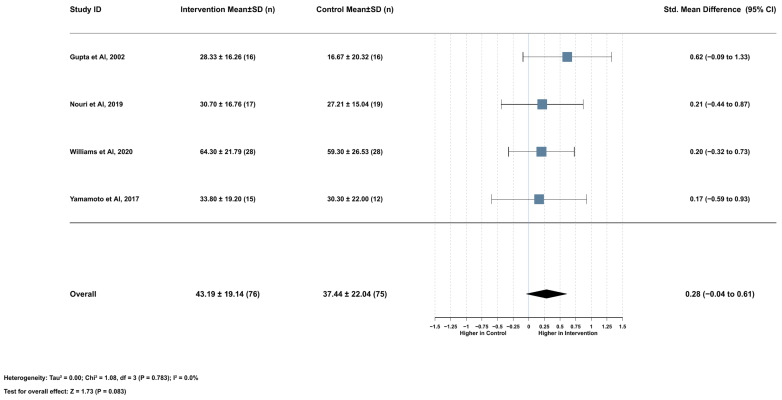
Lycopene supplementation effect on total sperm motility [16,17,18,19].

For total sperm motility, lycopene supplementation showed a positive trend that approached but did not reach statistical significance (SMD = 0.28, 95% CI [−0.04 to 0.61], *p* = 0.083). This analysis incorporated four studies with 151 participants (76 in the intervention group and 75 in the control group). The heterogeneity assessment indicated low variation between studies (I^2^ = 0.0%), justifying the application of a fixed effects model (Figure 3).

Leave-one-out sensitivity analyses confirmed the stability of this borderline finding, with effect sizes ranging from SMD = 0.20 (95% CI [−0.16 to 0.56], *p* = 0.282) when removing Gupta et al. (2002) [19] to SMD = 0.33 (95% CI [−0.07 to 0.74], *p* = 0.108) when removing Williams et al. (2020) [16]. All sensitivity analyses maintained zero heterogeneity (I^2^ = 0.0%), further supporting the consistency of the observed trend (Supplementary Figure S1). The funnel plot for total motility SMD demonstrated good symmetry, and Egger’s test confirmed the absence of significant publication bias (z = 0.36, *p* = 0.721), suggesting that the borderline beneficial effect on total sperm motility was not substantially influenced by reporting bias (Supplementary Figure S2).

#### 2.3.3. Association Between Serum Lycopene and Nonprogressive Motility

**Figure 4:**
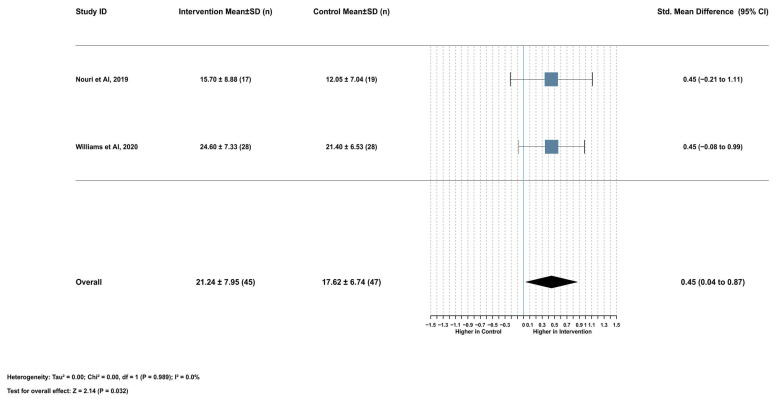
Lycopene supplementation’s effect on nonprogressive sperm motility [16,17].

Lycopene supplementation demonstrated a significant positive effect on nonprogressive sperm motility (SMD = 0.45, 95% CI [0.04 to 0.87], *p* = 0.032). This analysis included two studies with a combined 92 participants (45 in the intervention group and 47 in the control group). The heterogeneity assessment revealed low variation between the included studies (I^2^ = 0.0%), justifying the application of a fixed effects model. Mean nonprogressive motility values were 21.24 ± 7.95% in the intervention group versus 17.62 ± 6.74% in the control group, representing a mean difference of 3.34% (Figure 4). A comprehensive leave-one-out sensitivity analysis could not be performed because of the limited number of included studies, which represents a limitation in assessing the stability of this finding. Similarly, publication bias assessment via funnel plot was not feasible because of the insufficient number of studies.

#### 2.3.4. Association Between Serum Lycopene and Normal Morphology

**Figure 5:**
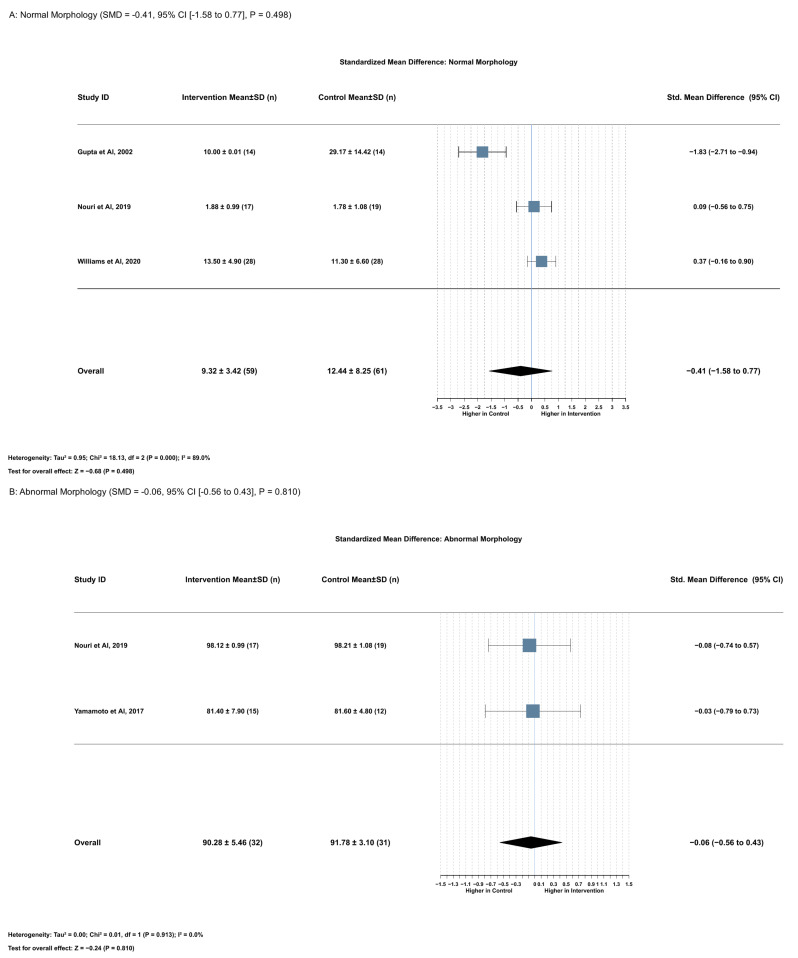
Lycopene supplementation’s effects on (**A**) normal sperm morphology and (**B**) abnormal sperm morphology [16,17,18,19].

Lycopene supplementation had no significant effect on normal sperm morphology (SMD = −0.41, 95% CI [−1.58 to 0.77], *p* = 0.498). This analysis included three studies with 120 participants (59 in the intervention group and 61 in the control group). Notably, heterogeneity was high (I^2^ = 89.0%), necessitating the use of a random effects model (Figure 5A).

Leave-one-out sensitivity analyses revealed substantial instability in this finding, with dramatic changes in effect size and direction across different permutations. When removing Gupta et al. (2002) [19], the effect size reversed direction and heterogeneity disappeared (SMD = 0.26, 95% CI [−0.15 to 0.67], *p* = 0.210, I^2^ = 0.0%), suggesting this study exerted considerable influence on the pooled result. When removing Williams et al. (2020) [6] or Nouri et al. (2019) [17], the negative effect strengthened (SMD = −0.84 and −0.70, respectively) with persistently high heterogeneity (I^2^ = 91.5% and 94.3%, respectively) (Supplementary Figure S1). The funnel plot for normal morphology SMD exhibited marked asymmetry, and Egger’s test confirmed significant publication bias (z = −3.49, *p* < 0.001) (Supplementary Figure S2).

#### 2.3.5. Association Between Serum Lycopene and Abnormal Sperm Morphology

Lycopene supplementation had no significant effect on abnormal sperm morphology (SMD = −0.06, 95% CI [−0.56 to 0.43], *p* = 0.810). This analysis included two studies with 63 participants (32 in the intervention group and 31 in the control group). The heterogeneity assessment demonstrated low variation between studies (I^2^ = 0.0%), supporting the use of a fixed effects model. Mean abnormal morphology percentages were 90.28 ± 5.46% in the intervention group compared with 91.78 ± 3.10% in the control group, representing a mean difference of −0.09% (95% CI [−0.76 to 0.58], *p* = 0.787) (Figure 5B). Comprehensive leave-one-out sensitivity analysis and publication bias assessment via funnel plot could not be performed because of the limited number of included studies.

#### 2.3.6. Association Between Serum Lycopene and DNA Damage

**Figure 6:**
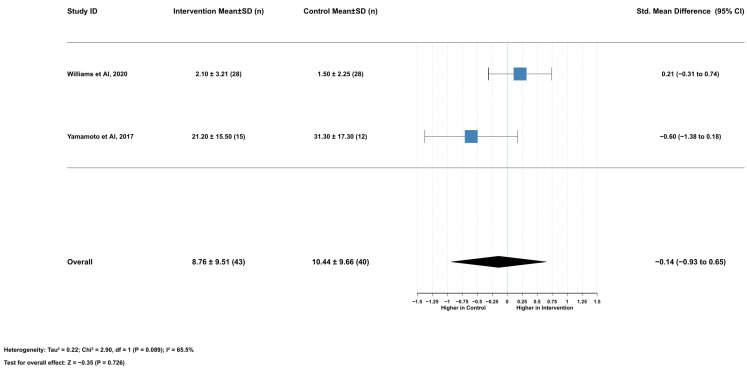
Lycopene supplementation’s effect on sperm DNA damage [16,18].

Lycopene supplementation had no significant effect on sperm DNA damage (SMD = −0.14, 95% CI [−0.93 to 0.65], *p* = 0.726). This analysis included two studies with 83 participants (43 in the intervention group and 40 in the control group). Heterogeneity was moderate to high (I^2^ = 65.5%), necessitating the use of a random effects model. Mean DNA damage percentages were 8.76 ± 9.51% in the intervention group versus 10.44 ± 9.66% in the control group, representing a mean difference of −2.86% (95% CI [−12.67 to 6.95], *p* = 0.568). The two included studies showed conflicting results: Williams et al. (2020) [16] reported a positive effect (SMD = 0.21, 95% CI [−0.31 to 0.74]), while Yamamoto et al. (2017) [18] reported a negative effect (SMD = −0.60, 95% CI [−1.38 to 0.18]) (Figure 6).

Comprehensive leave-one-out sensitivity analysis and publication bias assessment could not be performed because of the limited number of studies included.

#### 2.3.7. Association Between Serum Lycopene and Semen Volume

**Figure 7:**
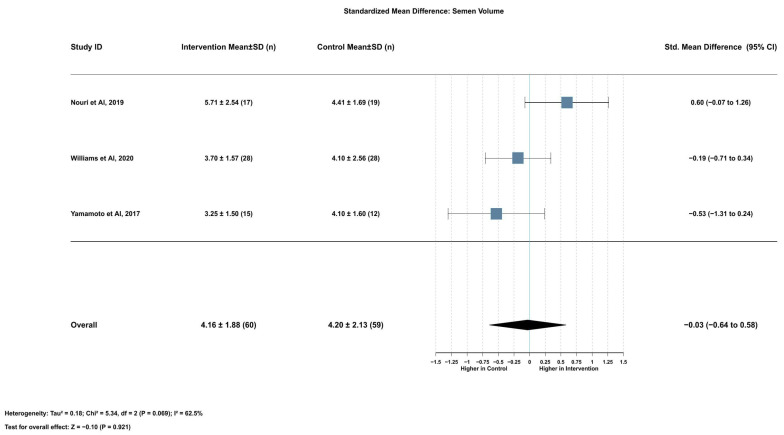
Lycopene supplementation’s effect on semen volume [16,17,18].

Lycopene supplementation had no significant effect on semen volume (SMD = −0.03, 95% CI [−0.64 to 0.58], *p* = 0.921). This analysis included three studies with 119 participants (60 in the intervention group and 59 in the control group). Heterogeneity was moderate to high (I^2^ = 62.5%), necessitating the use of a random effects model. Mean semen volume was 4.16 ± 1.88 mL in the intervention group versus 4.20 ± 2.13 mL in the control group, with a mean difference of −0.05 mL (95% CI [−1.23 to 1.13], *p* = 0.935) (Figure 7).

Leave-one-out sensitivity analyses revealed some instability in this finding. When removing Nouri et al. (2019) [17], which reported a positive effect, the pooled effect became more negative (SMD = −0.30, 95% CI [−0.73 to 0.14], *p* = 0.182) with heterogeneity reduced to I^2^ = 0.0%. When removing Yamamoto et al. (2017) [18] or Williams et al. (2020) [16], both of which reported negative effects, the pooled effect shifted toward positive (SMD = 0.18 and SMD = 0.05, respectively) with substantial remaining heterogeneity (Supplementary Figure S1). The funnel plot for semen volume SMD showed reasonable symmetry, but the limited number of studies precluded definitive assessment of publication bias (Supplementary Figure S2).

#### 2.3.8. Association Between Serum Lycopene and Nonmotile Sperm

**Figure 8:**
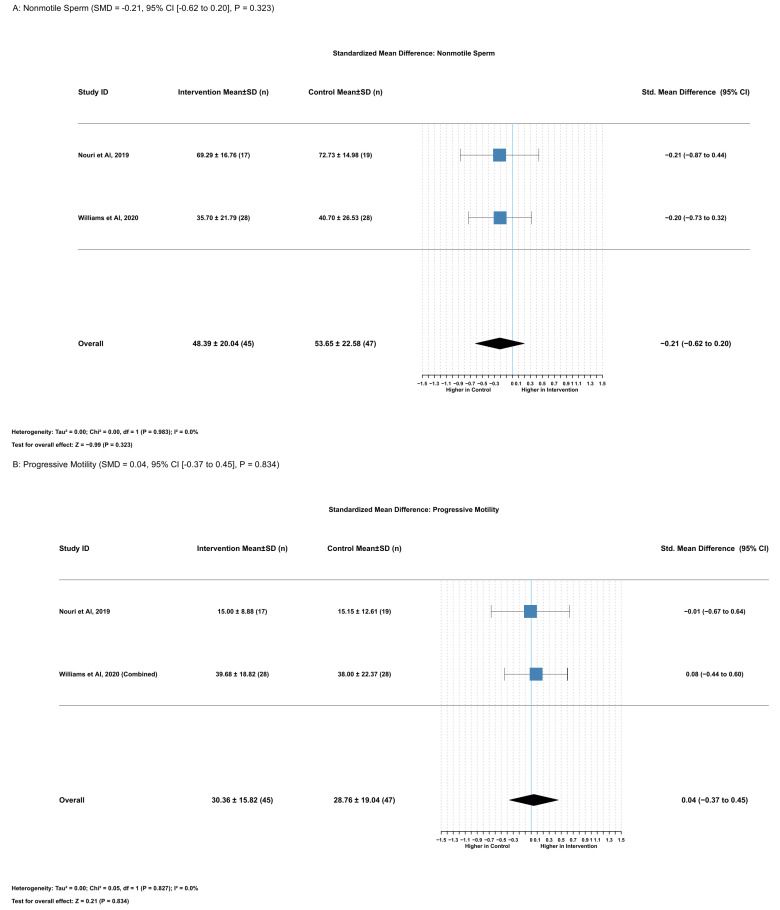
Lycopene supplementation’s effects on (**A**) nonmotile sperm percentage and (**B**) progressive motility [16,17].

Lycopene supplementation had no significant effect on nonmotile sperm percentage (SMD = −0.21, 95% CI [−0.62 to 0.20], *p* = 0.323). This analysis included two studies with 92 participants (45 in the intervention group and 47 in the control group). The heterogeneity assessment demonstrated low variation between studies (I^2^ = 0.0%), supporting the use of a fixed effects model. Mean nonmotile sperm percentages were 48.39 ± 20.04% in the intervention group versus 53.65 ± 22.58% in the control group, representing a mean difference of −4.07% (95% CI [−12.13 to 4.00], *p* = 0.323) (Figure 8A).

Both included studies showed similar direction and magnitude of effect: Nouri et al. (2019) [17] reported SMD = −0.21 (95% CI [−0.87 to 0.44]) and Williams et al. (2020) [16] reported SMD = −0.20 (95% CI [−0.73 to 0.32]). Comprehensive leave-one-out sensitivity analysis and publication bias assessment could not be performed because of the limited number of studies included.

#### 2.3.9. Association Between Serum Lycopene and Progressive Motility

Lycopene supplementation had no significant effect on progressive motility (SMD = 0.04, 95% CI [−0.37 to 0.45], *p* = 0.834). This analysis included two studies with 92 participants (45 in the intervention group and 47 in the control group). The heterogeneity assessment revealed low variation between studies (I^2^ = 0.0%), justifying the application of a fixed effects model. Mean progressive motility values were 30.36 ± 15.82% in the intervention group versus 28.76 ± 19.04% in the control group, representing a mean difference of 0.40% (95% CI [−5.52 to 6.32], *p* = 0.895). Both included studies showed similar minimal effects: Nouri et al. (2019) [17] reported SMD = −0.01 (95% CI [−0.67 to 0.64]) and Williams et al. (2020) [16] reported SMD = 0.08 (95% CI [−0.44 to 0.60]) (Figure 8B). Comprehensive leave-one-out sensitivity analysis and publication bias assessment were not feasible because of the insufficient number of studies.

#### 2.3.10. Risk of Bias Assessment

**Figure 9:**
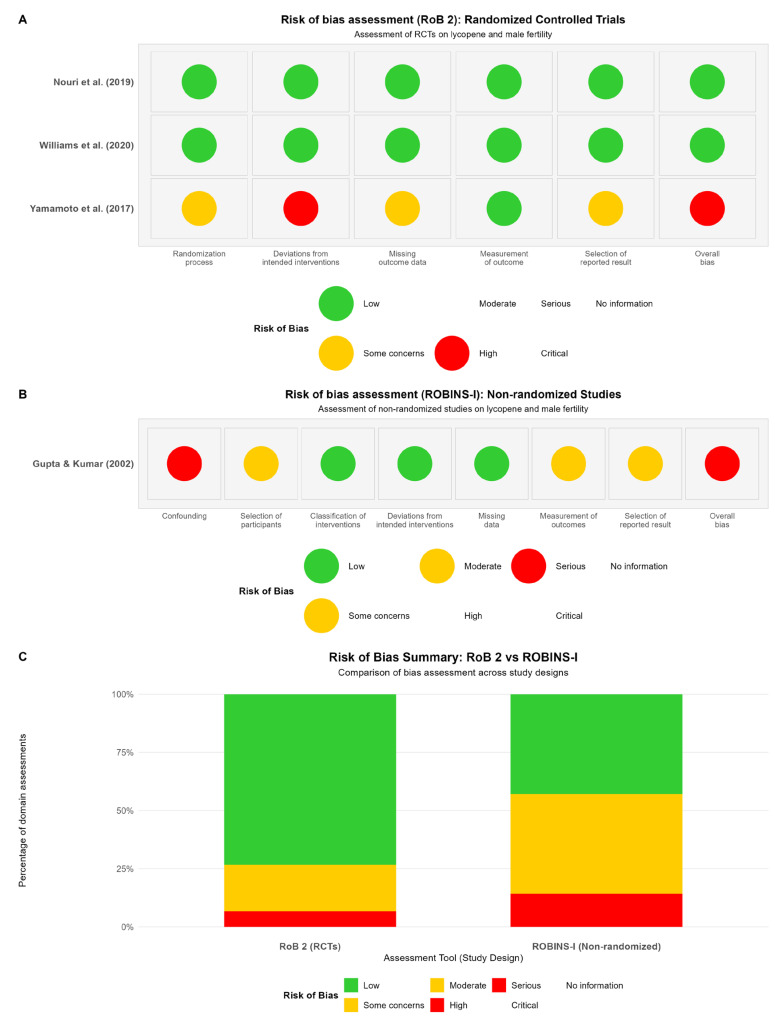
Risk of bias assessment for (**A**) randomized controlled trials and (**B**) nonrandomized studies. (**C**) Comparison of bias assessment [16,17,18,19].

The risk of bias assessment revealed considerable heterogeneity in study quality across the included evidence (Figure 9).

Among the three randomized controlled trials, two studies demonstrated low risk of bias across all domains [16,17]. Both studies employed appropriate randomization procedures, maintained double-blinding with identical placebo capsules, achieved complete or near-complete follow-up, used standardized outcome measurements, and reported pre-specified primary endpoints. In contrast, Yamamoto et al. (2017) exhibited high overall risk of bias, primarily due to the open-label design, which introduced performance and detection bias [18]. This study lacked blinding of participants, personnel, and outcome assessors and employed a no-intervention control group instead of a placebo control.

The single nonrandomized study was assessed using ROBINS-I and demonstrated a serious overall risk of bias [19]. The uncontrolled before–after design introduced substantial confounding bias, as the study lacked a comparison group to account for natural variation in semen parameters, regression to the mean, or temporal trends. While the study achieved complete follow-up and used standardized semen analysis methods, the fundamental design limitation severely compromised the ability to establish causal relationships between lycopene supplementation and observed improvements in sperm parameters.

The risk of bias assessment highlighted that high-quality evidence from well-conducted randomized trials provides the most reliable basis for evaluating lycopene’s effectiveness, while evidence from studies with methodological limitations should be interpreted with considerable caution [16,17].

## 3. Discussion

This systematic review and meta-analysis evaluated the effects of lycopene supplementation on key male fertility parameters, including sperm concentration, motility, morphology, DNA integrity, and seminal volume. In the four clinical studies analyzed, which included 151 participants, we found a statistically significant improvement in sperm concentration and nonprogressive motility after lycopene supplementation. The remaining parameters showed no statistically significant effects.

The increase in sperm concentration reinforces the hypothesis that the antioxidant properties of lycopene can mitigate oxidative stress, which is one of the main mechanisms involved in sperm damage [20]. The low heterogeneity between studies and consistency in sensitivity analysis support the strength of this finding. The improvement in nonprogressive motility was also positive, although based on only two studies, which limits its generalization.

In contrast, total and progressive motility did not show statistically significant improvements, although the effect was generally favorable. The borderline significance for total motility and consistent direction across studies may indicate a potential benefit that could be clarified in larger trials. Sperm morphology, both normal and abnormal, showed no significant effect and was associated with substantial heterogeneity and publication bias. This variability likely reflects methodological differences across studies and highlights the complexity of interpreting morphological outcomes in clinical trials in the future.

Similarly, semen volume, DNA damage, and nonmotile sperm percentage were not significantly affected by lycopene supplementation. These outcomes demonstrated moderate to high heterogeneity or were limited by the small number of studies included, making definitive conclusions difficult. Interestingly, although DNA damage is a critical endpoint reflecting sperm integrity [21], the conflicting directions of effect between studies and limited sample size undermine confidence in this result.

Williams et al. (2020) found that while lycopene supplementation did not lead to a statistically significant improvement in the concentration of motile sperm, it did significantly improve two key sperm quality parameters: the proportion of sperm with normal morphology and the proportion of fast-progressive sperm [16]. These findings suggest that lycopene may enhance certain aspects of sperm quality, which could be beneficial for infertile men. In addition, Williams et al. (2020) showed that lycopene supplementation did not affect sperm DNA damage, which is expected given ongoing debates about how best to measure and interpret this parameter [16].

The mechanisms underlying the observed findings suggest that lycopene may influence mitochondrial function, as indicated by some studies [22,23]. This potential effect on mitochondria is particularly significant, given that in sperm cells, mitochondria are concentrated in the intermediate position and play a crucial role in ATP production, which is essential for the proper movement of flagella. Mitochondria would imply more available energy and, therefore, an improvement in sperm motility. This hypothesis was bot directly evaluated in the studies analyzed; therefore, it cannot be assumed as the main mechanism. To date, most studies on lycopene have focused on its unique antioxidant capacity as the main mechanism of action and justification for use in different interventions. Although total antioxidant capacity in semen was not directly measured, the beneficial effects observed in this meta-analysis may be attributable to lycopene’s antioxidant properties.

The optimal duration and dose of lycopene supplementation are still not well defined. The 12-week choice in most trials is based on the average time required for spermatogenesis (63–70 days), which makes it the minimum necessary time, although it has not been reported that greater durations may exert more marked effects. Although there is no defined standard dose, the results of the present study coincide with those of previous studies that also showed improvements in motility and morphology with doses of between 4 and 14 mg daily, provided that they are ingested together with meals containing fat to improve their bioavailability.

Strengths and Limitations

The quality of evidence varied considerably across studies. While two randomized controlled trials were well-designed and exhibited low risk of bias, one trial and the single-arm study showed serious methodological concerns, including a lack of blinding and absence of control groups. These limitations may have introduced performance, detection, or confounding biases, potentially overestimating the benefits of lycopene intake. Nonetheless, our subgroup and sensitivity analyses generally supported the robustness of key findings and indicated minimal statistical heterogeneity for most outcomes. Taken together, these results suggest that lycopene may offer modest but meaningful benefits in improving certain semen parameters, particularly sperm concentration and nonprogressive motility. However, the lack of effect on other outcomes and the presence of methodological weaknesses in some studies highlight the need for larger, high-quality, placebo-controlled trials using standardized outcome measures to confirm these findings.

Future research should aim to define optimal dosing strategies, evaluate long-term effects, and explore whether lycopene supplementation is more effective in specific subgroups of infertile men, such as those with elevated oxidative stress or idiopathic infertility, than in others. Additionally, mechanistic studies could help elucidate how lycopene interacts with hormonal and oxidative pathways involved in spermatogenesis. This meta-analysis highlights that lycopene improves sperm concentration and nonprogressive motility but has limited effects on DNA damage, emphasizing the need for targeted antioxidants to protect sperm DNA integrity. As sperm DNA fragmentation strongly predicts fertility outcomes, identifying antioxidants with enhanced DNA-protective properties is essential. Future studies should explore combination therapies (e.g., lycopene with vitamin E, selenium, or N-acetylcysteine) for synergistic effects and use advanced DNA integrity tests, such as SCSA, TUNEL, and comet assays, as primary endpoints. Personalized antioxidant strategies based on oxidative biomarkers and genetic polymorphisms may help optimize outcomes. Additionally, determining the optimal timing, dosage, and duration of antioxidant use in relation to assisted reproduction is vital for maximizing DNA protection.

## 4. Materials and Methods

### 4.1. Study Protocol and Registration

To conduct this systematic review and meta-analysis [24], we used the Preferred Reporting Items for Systematic Reviews and Meta-Analysis (PRISMA). We followed all the steps mentioned in Cochrane’s Handbook of Systematic Reviews of Interventions [25].

### 4.2. Search Strategy and Data Collection

We searched four electronic databases: Scopus, PubMed, Web of Science (WoS), and Medline via WOS. We searched for all studies published until February 2025. We used the following terms: ((lycopene OR “lycopene supplementation” OR “lycopene extract” OR “dietary lycopene”) AND (prostatic OR prostate OR “semen analysis” OR semen OR “seminal fluid” OR fertility OR “sperm quality” OR spermatogenesis OR “sperm parameters” OR testosterone OR andrology OR “reproductive health” OR “male infertility” OR “Reproductive Health” OR “Prostatic Neoplasms” OR “Prostate” OR andrology OR “semen analysis” OR fertility OR “seminal fluid” OR spermatogenesis OR “sperm parameters” OR testosterone OR “male reproductive health” OR “seminal vesicles” OR “Vas deferens”)). We included all types of primary studies except case reports and case series that assessed the use of lycopene in male fertility health. We included only English studies between 2001 and 2025.

We removed duplicates using EndNote Software Version (X-9). We assessed all the retrieved studies for our eligibility criteria in two steps. First, we conduced title and abstract screening; then, we screened the full text of the remaining retrieved studies. Studies that met our eligibility criteria were included. Two separate authors performed all of the screening steps. A third author solved conflicts.

### 4.3. Data Extraction and Outcome Measurements

We extracted data using Excel in two sheets, a summary and baseline sheet and an outcome sheet, by two separate authors; the third author solved conflicts.

### 4.4. Quality Assessment

We evaluated the methodological rigor of our included studies using two distinct tools. RCTs underwent assessment via the Cochrane RoB 2 framework [26]. This approach examines potential bias across five key areas: randomization procedures (D1), protocol adherence (D2), data completeness (D3), outcome measurement (D4), and selective reporting (D5). Our team assigned “Low risk”, “Some concerns”, or “High risk” ratings to each domain, then derived comprehensive judgments for each study as recommended by Higgins and colleagues [27].

Nonrandomized investigations required a different approach; we selected the ROBINS-I instrument [28] for these assessments. This more nuanced tool scrutinizes seven potential bias sources: confounding variables (D1), participant selection methods (D2), intervention classification (D3), protocol deviations (D4), missing information (D5), outcome measurement techniques (D6), and selective result reporting (D7). For these studies, we applied “Low”, “Moderate”, “Serious”, or “Critical” risk designations across domains, with the most concerning rating determining the overall assessment, following established guidance [29].

Two team members independently conducted these evaluations, comparing their findings afterward. Whenever disagreements emerged, we held thorough discussions and occasionally consulted a third colleague to break stalemates. While no studies faced exclusion based solely on these quality metrics, our risk assessments substantially shaped our interpretation of findings and informed our sensitivity analysis strategy under current best practices [30].

### 4.5. Data Analysis

Statistical analyses were performed using R software (v. 4.4.1) with the ‘meta’, ‘metafor’, and ‘forestplot’ packages. Standardized mean difference (SMD) was calculated using Hedges’s g with small sample correction to account for different measurement scales across studies. The DerSimonian and Laird random effects model was employed to incorporate both within-study and between-study variance in the meta-analysis. Forest plots were generated to visualize SMD effect estimates and corresponding 95% confidence intervals (CIs) for each outcome. To assess the robustness of findings, leave-one-out sensitivity analyses were conducted by systematically removing one study at a time and recalculating the pooled effect. Publication bias was evaluated using funnel plots and Egger’s regression test for funnel plot asymmetry. Statistical significance was defined as *p* < 0.05, with positive SMD values indicating higher levels in the intervention group compared with the control group [31,32,33,34].

## 5. Conclusions

This systematic review and meta-analysis provides encouraging evidence that lycopene supplementation may modestly improve sperm concentration and nonprogressive motility in men. However, lycopene showed no significant effect on other semen parameters, including progressive motility, morphology, semen volume, and DNA damage. The improvements detected in key markers of male fertility are clinically relevant, especially considering the high global burden of fertility problems. These findings support the potential of lycopene as a personalized nutritional supplement for male reproductive health. These findings suggest the potential but limited benefits of lycopene in supporting male fertility. Future well-designed randomized controlled trials with standardized outcome measures and larger sample sizes are needed to establish definitive clinical recommendations.

## Figures and Tables

**Table 1:** Summary of the characteristics of the included studies and patients.

Study ID	Setting (Country)	Study Design	Sample Size		Condition of the Included Patients (PC/BPH/Healthy)		Form of Intervention		Duration of Follow-Up	Baseline Data										
			Intervention	Controls	Intervention	Controls	Lycopene Supplement (dose)	Control		Age (Mean ± SD)/Age Group, Years		Smoking Status (%)		BMI Mean (sd)		% of Body Fat		No. of Intervention Days		Total Lycopene Consumed During the Study, mg/day
										Intervention	Control	Intervention	Control	Intervention	Control	Intervention	Control	Intervention	Control	Intervention
Williams et al., 2020 [16]	UK	Randomized, double-blind, placebo-controlled clinical trial	28	28	Healthy	Healthy	7 mg twice/day	Placebo	3 months	23.4 ± 3.22	23.3 ± 2.58	14.30%	14.30%	25.2 ± 3.08	23.5 ± 3.11	NA	NA	3 months	3 months	14 mg/day
Nouri et al., 2019 [17]	Iran	Randomized, double-blind, placebo-controlled clinical trial	17	19	Primary/secondary infertility	Primary/secondary infertility	25 mg once a day and a second	Placebo	3 months	32.89 ± 2.33	32.15 ± 2.16	6 (35.29%)	8 (42.1%)	26.94 ± 1.57	26.53 ± 1.53	28.35 ± 3.23	27.98 ± 3.69	3 months	3 months	25 mg/day and a second
Yamamoto et al., 2017 [18]	Japan	Randomized clinical trial	17	12	Infertility	Infertility	30 mg once/day	NA	3 months	38.1 ± 1.76	36.2 ± 1.91	NA	NA	NA	NA	NA	NA	3 months	3 months	30 mg
Gupta et al., 2002 [19]	India	Single arm clinical trial	30		Idiopathic infertility		2000 mcg twice/day		3 months		NA		NA		NA		NA		NA	

NA: not assessed, BMI: body mass index.

## Data Availability

All data generated or analyzed during this study are included in this published article [and its Supplementary Information files].

## Supplementary Materials

The following supporting information can be downloaded at: https://www.mdpi.com/article/10.3390/ijms26157224/s1.

## Abbreviations

The following abbreviations are used in this manuscript:

SMDs	Standardized mean differences
