# Supplementary material for: Association of Lycopene and Male Reproductive Health: Systematic Review and Meta-Analysis
Source: Int J Mol Sci. 2025 Jul 25;26(15):7224. doi: 10.3390/ijms26157224 (PMC12346668; doi:10.3390/ijms26157224)
Supplement: Supplementary file 1 [file ijms-26-07224-s001.zip › Supplementary Figure S1.pdf]

A: Sperm Concentration

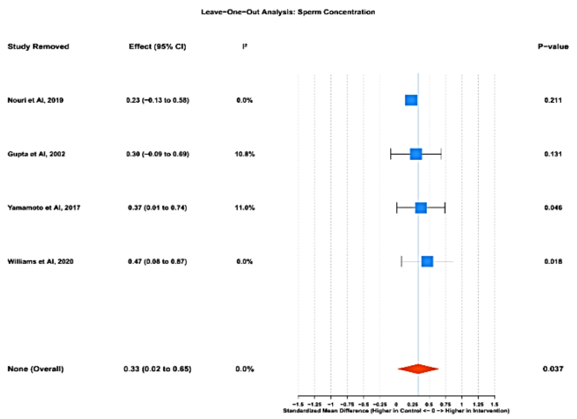

B: Total Motility

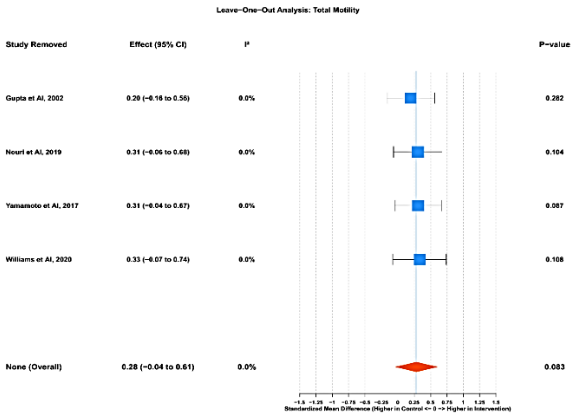

C: Normal Morphology

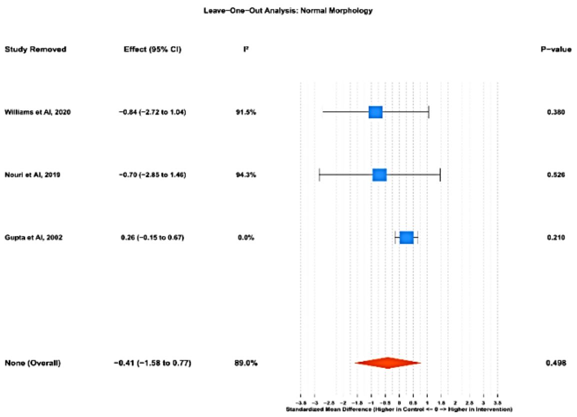

D: Semen Volume

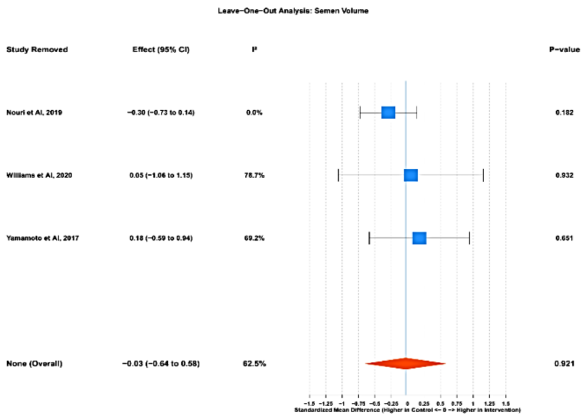

Supplementary Figure S1. Leave-one-out analysis of (a) sperma concentration, (b) motility,(c) normal morphology and (d) semen volume [16-19]
