# Supplementary material for: Association of Lycopene and Male Reproductive Health: Systematic Review and Meta-Analysis
Source: Int J Mol Sci. 2025 Jul 25;26(15):7224. doi: 10.3390/ijms26157224 (PMC12346668; doi:10.3390/ijms26157224)
Supplement: Supplementary file 1 [file ijms-26-07224-s001.zip › Supplementary Figure S2.pdf]

A: Sperm Concentration (Egger's test: z = 0.71, p = 0.481)

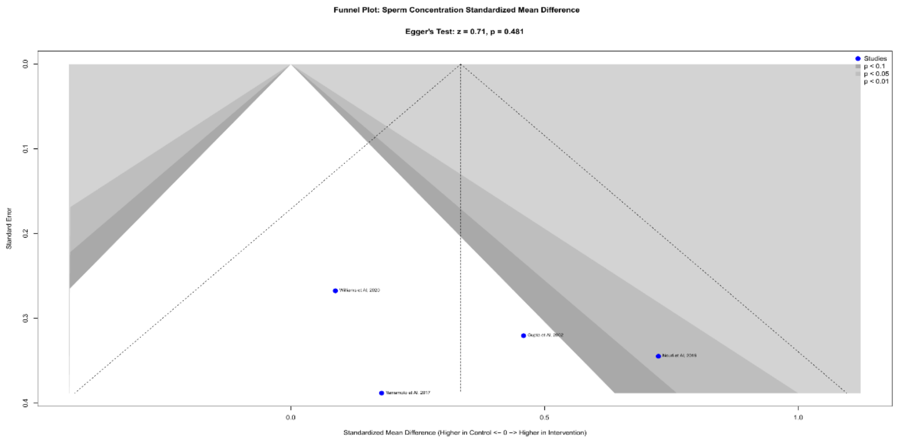

B: Total Motility (Egger's test: z = 0.36, p = 0.721)

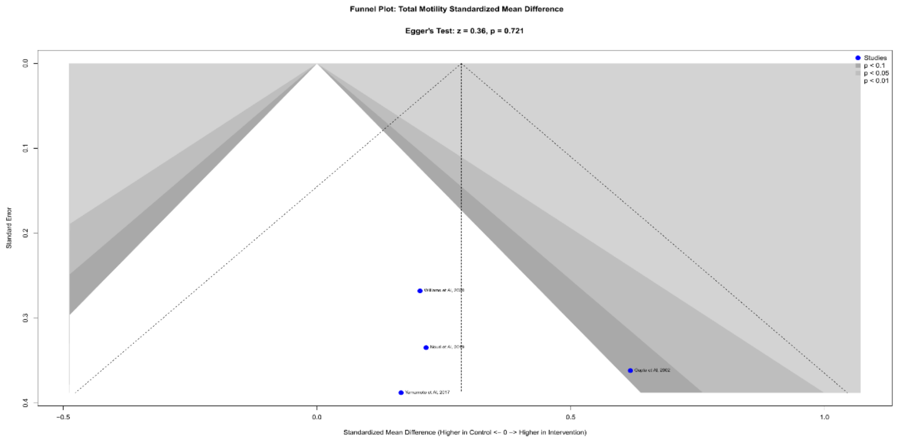

C: Normal Morphology (Egger's test: z = -3.49, p < 0.001)

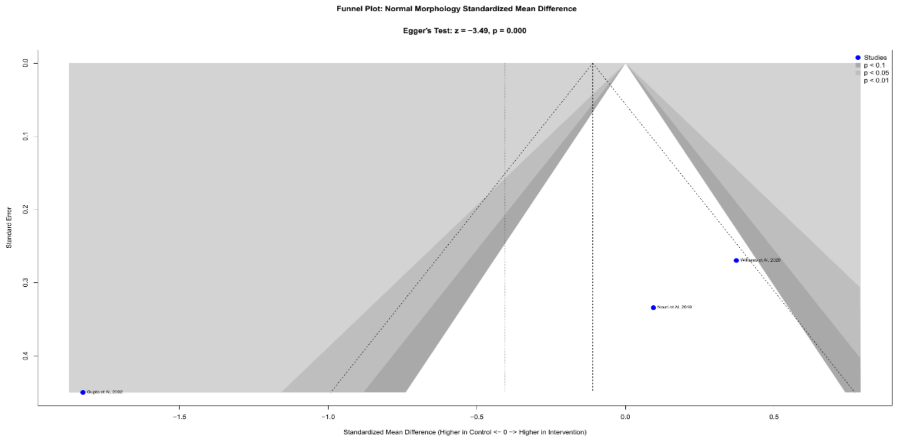

D: Semen Volume (Egger's test: z = -0.18, p = 0.857)

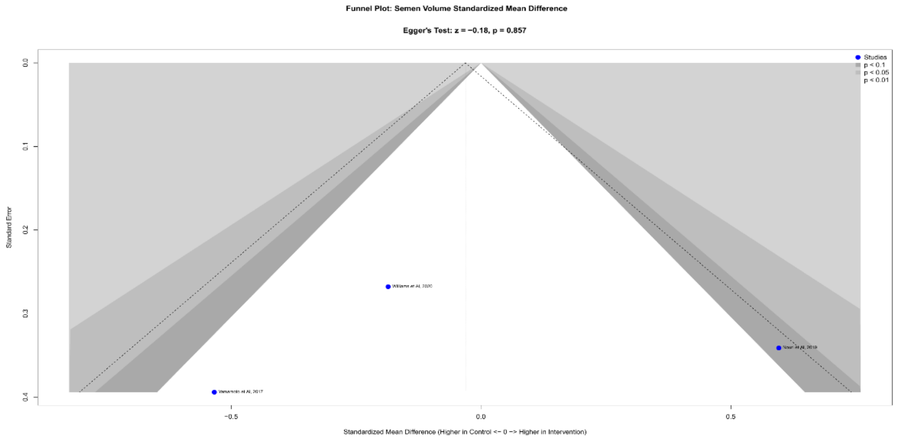

Supplementary Figure S2. Funnel Plots of (a) sperm concentration, (b) total motility, (c) normal morphology and (d) semen volume
