# Supplementary figures and images for: Association of Lycopene and Male Reproductive Health: Systematic Review and Meta-Analysis
Source: Int J Mol Sci. 2025 Jul 25;26(15):7224. doi: 10.3390/ijms26157224 (PMC12346668; doi:10.3390/ijms26157224)

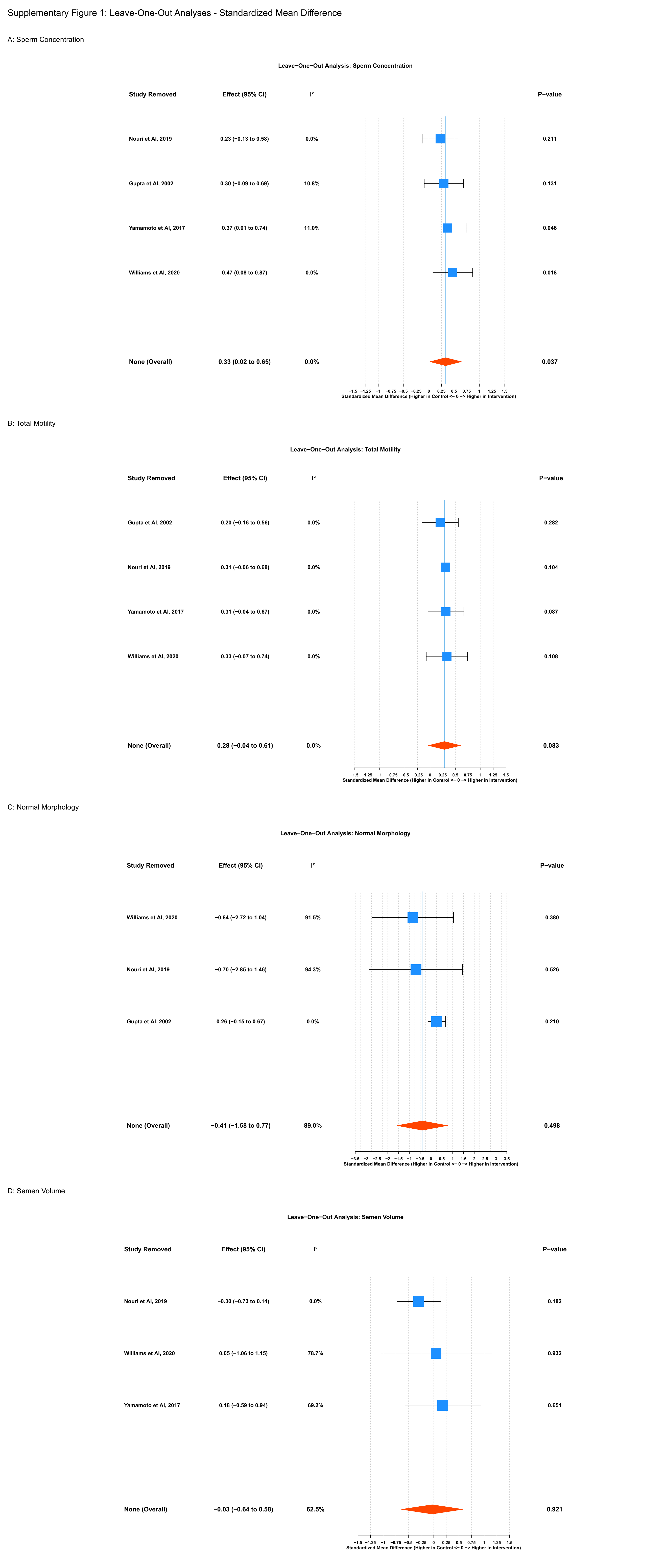

Supplement: Supplementary file 1 [file ijms-26-07224-s001.zip › SuppFigure1_LOO_SMD.png]

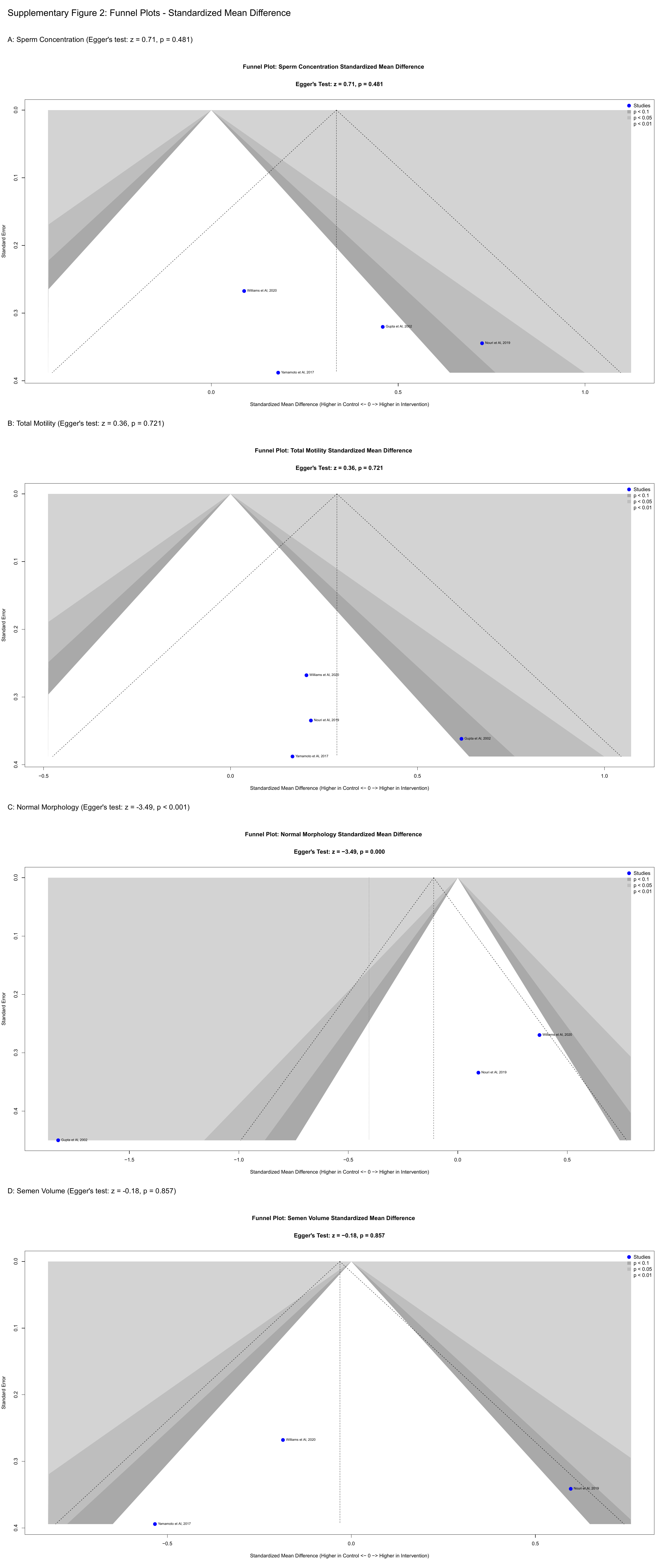

Supplement: Supplementary file 1 [file ijms-26-07224-s001.zip › SuppFigure2_Funnel_SMD.png]
